# Supplementary material for: Transcriptomic Analysis of Inbred Chicken Lines Reveals Infectious Bursal Disease Severity Is Associated with Greater Bursal Inflammation In Vivo and More Rapid Induction of Pro-Inflammatory Responses in Primary Bursal Cells Stimulated Ex Vivo
Source: Viruses. 2021 May 18;13(5):933. doi: 10.3390/v13050933 (PMC8157851; doi:10.3390/v13050933)
Supplement: Supplementary file 1 [file viruses-13-00933-s001.zip › viruses-1208183-supp/viruses-1208183-Table S5.pdf]

Table S5. Primers used in this study.

Name	Forward Sequence (5′-3′)	Reverse Sequence (5′-3′)
IBDV	GAGGTGGCCGACCTCAACT	GCCCGGATTATGTCTTTGAAG
IL-1β	GCTCTACATGTCGTGTGATGAG	TGTCGATGTCCCGCATGA
IL-6	AACATGCGTCAGCTCCTGAAT	TCTGCTAGGACTTCTCCATTGAA
IL-8	GCCCTCCTCCTGGTTTCAG	TGGCACCGCAGCTCATT
iNOS	CCTGGAGGTCCTGGAAGAGT	CCTGGGTTTCAGAAGTGGC
